# Cathodic voltage-controlled electrical stimulation and betadine decontaminate nosocomial pathogens from implant surfaces

**DOI:** 10.1128/msphere.00583-23

**Published:** 2024-02-01

**Authors:** Tripti Thapa Gupta, Bernadette Zumpano, Jacob Opalinski, Riley Ritchey, Nathan Winter, Scott R. Nodzo, Mary Canty

**Affiliations:** 1 Garwood Medical Devices, LLC, Buffalo, New York, USA; 2 Department of Orthopedics & Sports Medicine, University at Buffalo, Buffalo, New York, USA; College of Medicine, University of Nebraska Medical Center, Omaha, Nebraska, USA

**Keywords:** biofilm, orthopedic infections, electrical stimulation, cobalt chromium, betadine

## Abstract

Periprosthetic joint infection (PJI) after total joint arthroplasty is a major concern requiring multiple surgeries and antibiotic interventions. *Staphylococcus aureus*, *Pseudomonas aeruginosa*, and *Escherichia coli* are the predominant causes of these infections. Due to biofilm formation, antibiotic treatment for patients with PJI can prolong resistance, further complicating the use of current treatments. Previous research has shown that cathodic voltage-controlled electrical stimulation (CVCES) is an effective technique to prevent/treat implant-associated biofilm infections on titanium (Ti) surfaces. This study thus evaluated the efficacy of CVCES via the use of 10% betadine alone and in combination with CVCES to eradicate lab-grown biofilms on cemented and cementless cobalt–chromium (CoCr) and Ti surfaces. CVCES treatment alone for 24 hours demonstrated no detectable CFU for *E. coli* and *P. aeruginosa* biofilms on cementless CoCr implants. In the presence of cement, *E. coli* biofilms had 10^6^ CFUs/implant remaining after CVCES treatment alone; however, *P. aeruginosa* biofilms on cemented implants were reduced to below detectable limits. The use of 10% betadine treatment for 3 minutes followed by 24-hour CVCES treatment brought CFU levels to below detectable limits in *E. coli* and *P. aeruginosa*. The same was true for *S. aureus* biofilms on cementless patellofemoral implants as well as femoral and tibial implants. These treatment methods were not sufficient for eradication of *S. aureus* biofilms on cemented implants. These results suggest that CVCES alone and CVCES with 10% betadine are effective approaches to treating biofilms formed by certain bacterial species potentially leading to the treatment of PJI.

**IMPORTANCE:**

Periprosthetic joint infections (PJIs) are problematic due to requiring multiple surgeries and antibiotic therapies that are responsible for increased patient morbidity and healthcare costs. These infections become resistant to antibiotic treatment due to the formation of biofilms on the orthopedic surfaces. Cathodic voltage-controlled electrical stimulation (CVCES) has previously been shown to be an effective technique to prevent and treat biofilm infections on different surfaces. This study shows that CVCES can increase the efficacy of 10% betadine irrigation used in debridement, antibiotics, and implant retention by 99.9% and clear infection to below detection limits. PJI treatments are at times limited, and CVCES could be a promising technology to improve patient outcomes.

## INTRODUCTION

Periprosthetic joint infection (PJI) is one of the most challenging complications of total joint arthroplasty and is the most common cause of revision for failed knee arthroplasty (16.8% to 25.2%) (1–3). The incidence of PJI remains between 1% and 3% (4) in primary and 3% and 10% in revision arthroplasty (5, 6). By the year 2030, the number of PJIs is expected to increase to almost 10,000 cases per year (6). Along with increasing hospital financial burden, PJI poses serious complications such as extended periods of hospitalization and re-operations. These infections are devastating to patients, with increased rates of mortality and morbidity (7, 8), decreased quality of life (9), and potential for decreased levels of mobility and limb ambulation (10, 11). Current treatment of PJI involves surgical intervention and treatment with antibiotics. Antibiotic treatment is often challenging during PJI as bacteria often exist in a biofilm state, which makes them thousands of times more resistant than planktonic cells (12, 13). Debridement, antibiotics, and implant retention (DAIR) is a treatment method for acute infections with a stable prosthesis that has a variable success rate between 14% and 100% (14). DAIR involves extensive debridement with removal of necrotic tissue, exchange of modular components, and irrigation of the infected joint (15). In the clinical scenario where DAIR is deemed inappropriate, one- and two-stage revision exchanges are utilized (14). Multiple intraoperative treatment strategies have been developed in an attempt to decrease infection rates; however, we have not eliminated this complication (16). And the likelihood of developing a recurrent infection is significantly increasing (6, 16).

Gram-positive bacteria such as staphylococci (*Staphylococcus aureus* and coagulase-negative staphylococci) and streptococci constitute 65%–85% of all PJI isolates, and Gram-negative bacteria constitute 6%–23% (17). Although infections by Gram-negative organisms make up a relatively minor proportion of all PJIs, their virulence and growing resistance to antimicrobial agents make their treatment more complicated than Gram-positive organisms (17–19). To date, no resistance to common surgical irrigants such as betadine has been reported (20), and their use could facilitate biofilm elimination. Betadine is an antiseptic and a disinfectant with antimicrobial activity against Gram-positive and Gram-negative organisms, anaerobic bacteria, fungi, and viruses (20). The povidone-iodine in the betadine releases free iodine in solution that penetrates bacterial cell membranes and interacts with proteins, nucleotides, and fatty acids in the cytoplasm, resulting in cell death (21, 22). A 10% povidone-iodine solution is commonly used as a surgical scrub pre- and post-operatively to surgical sites (23, 24) and has been used intraoperatively during the DAIR procedure (25).

As a result of difficulties treating bacterial biofilms and the rise in drug-resistant bacteria, alternative therapeutic strategies must be investigated. Cathodic voltage-controlled electrical stimulation (CVCES) has been shown to be a promising technique to prevent and eradicate implant-associated methicillin-resistant *S. aureus* (MRSA) infections (26, 27). To further investigate the potential of CVCES as a novel therapeutic modality for the eradication of biofilms formed by multidrug-resistant pathogens, this *in vitro* study evaluated the antimicrobial efficacy of CVCES alone and in combination with 10% betadine against *S. aureus*, *Pseudomonas aeruginosa*, and *Escherichia coli* on commercially available orthopedic implants. Furthermore, the biofilm formation by these species was investigated in both cemented and cementless implants as both methods are used clinically (28). The role of bacteria living in and on cement during PJI has been previously evaluated, suggesting cement to be a nidus for bacteria, which may increase failure rates when cement is retained during treatment of a PJI (29, 30). Cementless total knee implants are gaining popularity, and cementless total hip replacement remains the mainstay of fixation in North America (31). Therefore, it is essential to investigate the efficacy of CVCES on biofilm formed in cemented and cementless implants. The primary goal of the present study was to evaluate the efficacy of CVCES alone and in combination with a 10% betadine solution to treat *S. aureus*, *P. aeruginosa*, and *E. coli* biofilms on cementless and cemented implants.

## MATERIALS AND METHODS

### Bacterial strains and culture conditions

Gram-positive MRSA (clinical strain CA127, NR-46172), Gram-negative *P. aeruginosa* (ATCC 27853), and *E. coli* (ATCC 25922) were used in this study. A single bacterial colony from tryptic soy agar (TSA) streaked plate was used to inoculate 10 mL of tryptic soy broth (TSB) for each experiment. Culture tubes were incubated statically at 37°C and 5% CO_2_ overnight.

### Patellofemoral and commercially available femoral and tibial implant preparation

**Fig 1:**
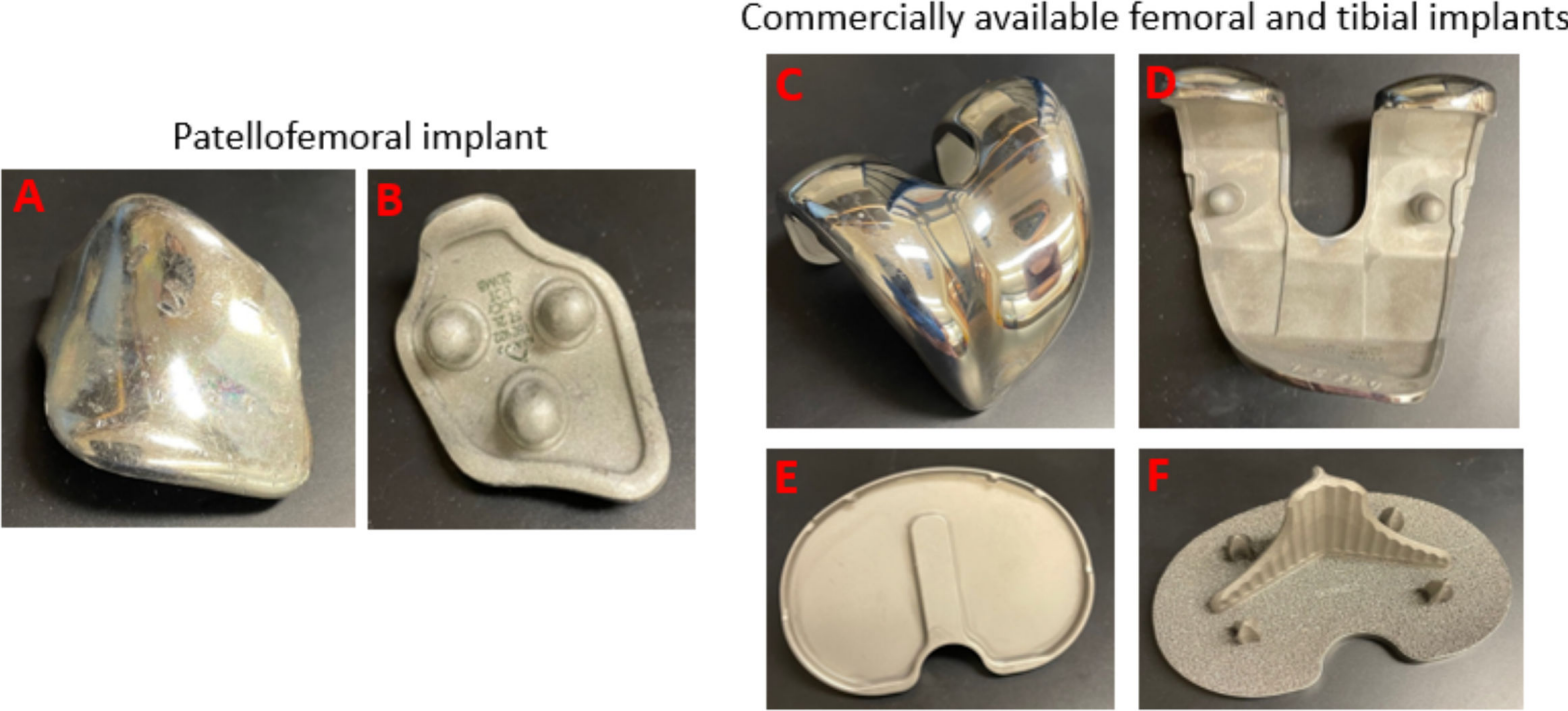
Photograph of patellofemoral and commercially available femoral and tibial implants. A, C, and E are the top and B, D, and F are the back views of the implants.

Stryker MAKO Restoris size 2 patellofemoral implant (Stryker, Mahwah, NJ, USA) and commercially available cobalt-chromium (CoCr) femoral and titanium alloy tibial implants (Fig. 1) with 3D-printed ingrowth surfaces were obtained. Implants were sterilized overnight in 70% ethanol and autoclaved on a dry cycle prior to use.

### Chamber preparation

**Fig 2:**
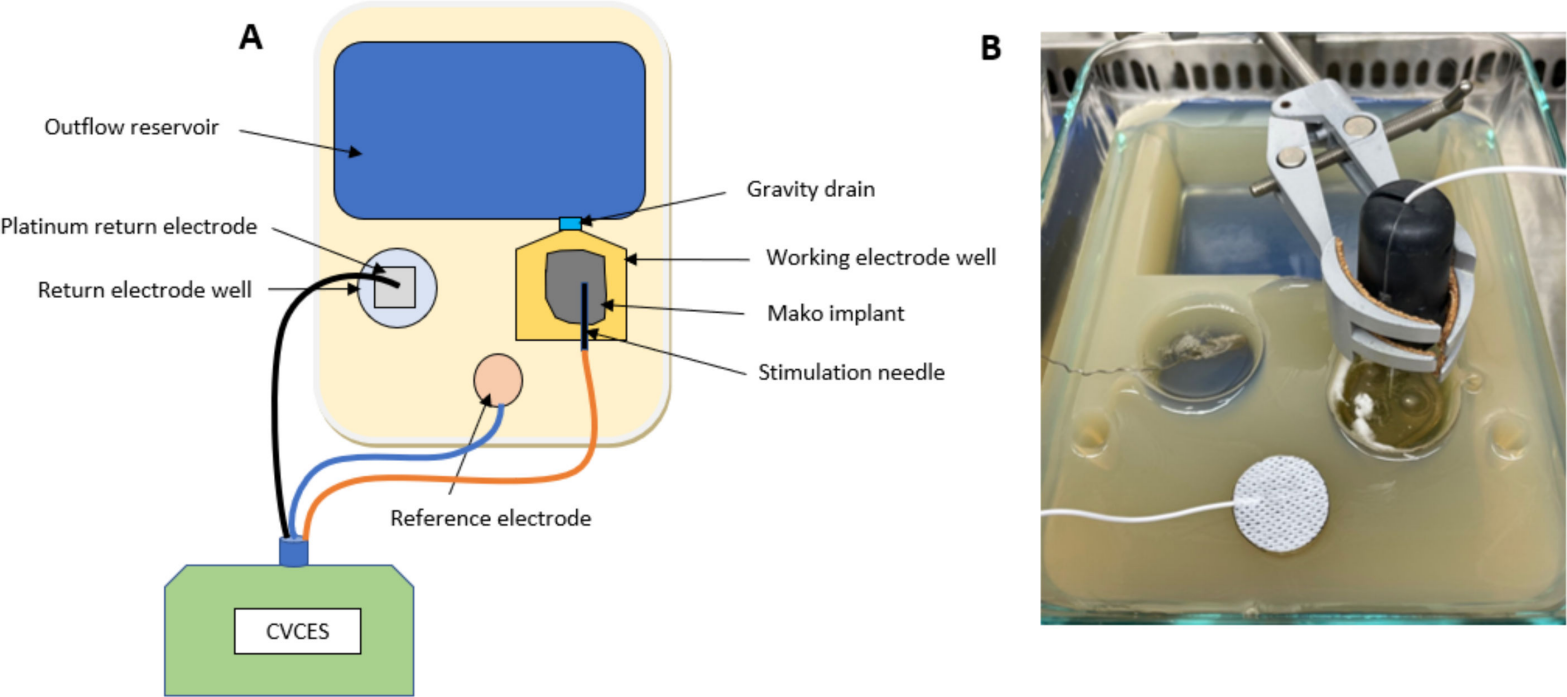
Schematic diagram (**A**) and photograph (**B**) of Pyrex electrochemical agar chamber for patellofemoral implant testing.

**Fig 3:**
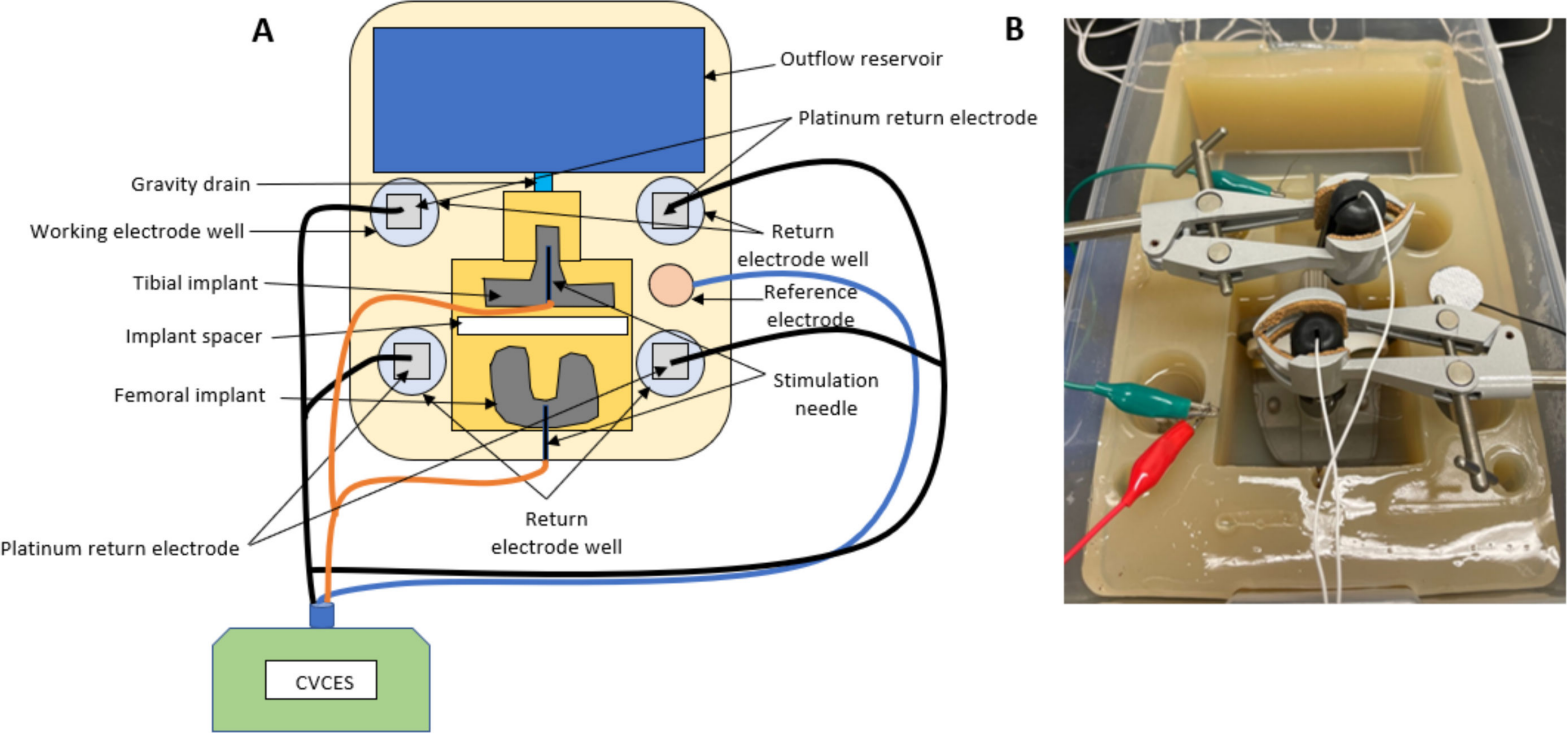
Schematic diagram (**A**) and photograph (**B**) of Pyrex electrochemical agar chamber for commercially available femoral and tibial implant testing.

Custom 3D-printed resin molds were placed in a sterile glass Pyrex dish (8″ × 6″ × 2″ for patellofemoral implant and 9″ × 13″ × 3.85″ for the femoral and tibial implants). Standard method agar (Cole Parmer EW-14200-32) (3%) in sterile saline (0.9% NaCl) was autoclaved and poured into the dish with molds. Upon cooling, molds were removed from the dish which left three distinct wells in the solidified agar (Fig. 2B and 3B). The completed chambers were stored at 4°C until ready for use. The agar chamber uses a standard three-electrode system with the patellofemoral and femoral and tibial implants as the working electrode, a platinum mesh as the return electrode, and a silver/silver chloride (Ag/AgCl) reference hydrogel electrode. An electrical connection was made to the working electrode with a Neuroline Monopolar needle (Ambu USA). All voltages are reported with respect to Ag/AgCl.

### *In vitro* biofilm growth assay

Bacterial strains of interest were struck out on TSA plates, allowed to incubate overnight at 37°C, 5% CO_2_, and then stored at 4°C until ready for use. A sterile inoculating wand was used to add one bacterial colony to 10 mL of TSB media, and the sample was incubated statically overnight at 37°C, 5% CO_2_. Sterilized Mako patellofemoral and commercially available femoral and tibial implants were placed in beakers and covered with TSB media supplemented with 10% human plasma by volume and 1% overnight culture by volume. The samples were then left to incubate for 48 hours at 37°C, 5% CO_2_. Following incubation, sterile forceps were used to remove and wash the implants with phosphate-buffered solution (PBS) to remove loosely adherent bacteria, before placing them in a new beaker and again covering with TSB media supplemented with 10% human plasma by volume. After an additional 24-hour incubation at 37°C, 5% CO_2_, the biofilms were prevalent on the implants and ready for experiments.

### Cemented implant preparation

**Fig 4:**
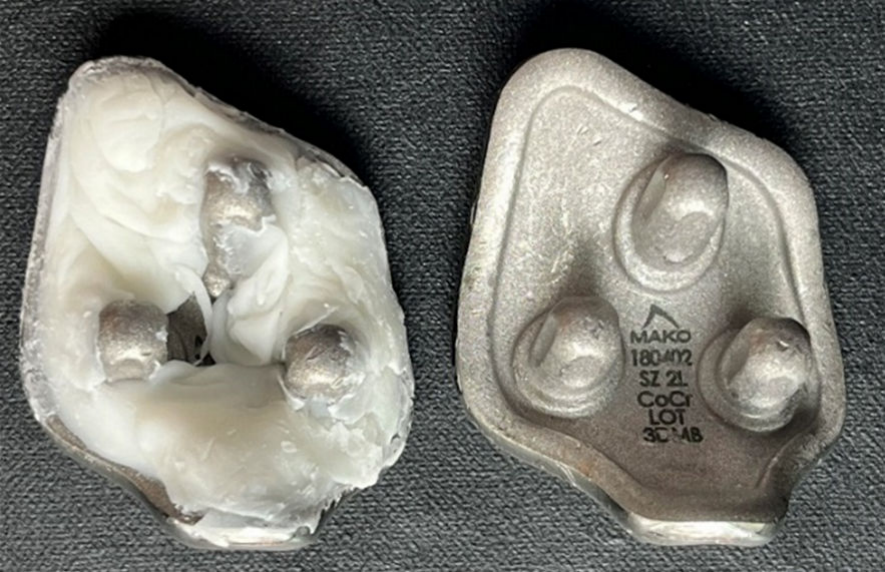
CoCr patellofemoral implant with and without cement.

Stryker surgical Simplex P radiopaque Polymethylmethacrylate (PMMA) bone cement was aseptically prepared by combining the powder and liquid components together in a sterile specimen cup under laminar flow. The cement was then hand-mixed with a sterile wooden spatula. Cement was added to the back of the sterile patellofemoral implant (Fig. 4) and allowed to solidify prior to use. The implants were then left for 1 hour to solidify in their cemented state before being used for biofilm preparation or stored in a sterile container until ready for use.

### CVCES treatment

The untreated and treated implants with 10% betadine were harvested immediately after growth incubation. The remaining implants were placed into flow chambers, covered with 25 mL of TSB and supplied with fresh TSB at 0.08 mL/min for the duration of the experiment, and treated with CVCES alone or a 3-minute betadine soak followed by CVCES (10% betadine + CVCES). A Neuroline Monopolar needle was used to make a connection to the implant that was connected directly to CVCES. Next to the working electrode well was a return electrode well where a platinum mesh electrode was covered with 25 mL of PBS and connected directly to the CVCES unit. Finally, the reference electrode was connected between the working well and the return well to complete the circuit to effectively run the CVCES treatment. Experiments were conducted at room temperature with the implants maintained at −1.9V vs Ag/AgCl for 24 hours to treat biofilms in the presence or absence of bone cement. The treatment time was chosen based on our past research, which showed a more robust antimicrobial effect after 24 hours of CVCES compared to 1 hour (27, 32).

### Bacterial enumeration

At the conclusion of the study, each implant was individually rinsed three times with sterile PBS. The samples were then immersed in 100 mL of 0.1% saponin and PBS solution. From there, the samples were vortexed for 1 minute, subjected to a 20-minute sonication, followed by another 1-minute vortex to dislodge bacteria attached to the surface. A 10-mL aliquot of the supernatant was then placed in a centrifuge tube and centrifuged for at least 20 minutes at 8,000 rpm. After the centrifuge cycle, bacterial pellets were resuspended in 5-mL PBS, and viable CFUs were enumerated via microdilution method by plating 10 µL of serial dilutions onto TSA plates and incubating at 37°C, 5% CO_2_, overnight.

### pH measurements

After the 24-hour treatment stimulation, a pH meter (SevenExcellence, Mettler Toledo) was used to measure the pH changes of the media surrounding the implant following the 24-hour incubation/stimulation protocol for all experiments. A pH measurement was taken for each sample treated with CVCES.

### Charge transfer calculations

Cathodic current measurements were collected for the duration of CVCES under all conditions. The cathodic current was integrated with respect to time to determine the total charge transferred during stimulation. Calculations were performed using the Riemann sum trapezoidal method to determine the cumulative charge transferred during CVCES.

### Qualitative SEM assessment of *S. aureus* and *P. aeruginosa* biofilms on implants

For scanning electron microscopy (SEM) images, implants with biofilm were fixed according to a procedure described previously (33). Implants were placed in a sterile container and soaked in a prefixing agent containing 2.5% glutaraldehyde in 0.2-M cacodylate buffer (pH 7.4) for 24  hours at room temperature. The implants were then rinsed with cacodylate buffer three times for 5 to 10 minutes. After the final rinse, the implants were dehydrated by placing them in increasing concentrations of ethanol (70%, 90%, and 100%) three times each for 5 minutes. Finally, the coupons were dried in a critical point dryer (Leica EM CPD300) and then viewed under an SEM (FE-SEM SU-70) at an accelerating voltage of 2 kV.

### Statistical analysis

All experiments in this study were repeated twice (two biological replicates). Prism (GraphPad v9.5.0 software) was used for all statistical analysis. The threshold for significance was set at a *P*-value of <0.05. Statistics presented are acquired via ordinary one-way analysis of variance with log-transformed CFU value followed by Tukey’s multiple comparisons test.

## RESULTS

### Eradication of biofilms using CVCES alone and in combination with 10% betadine in patellofemoral cemented and cementless implants

**Fig 5:**
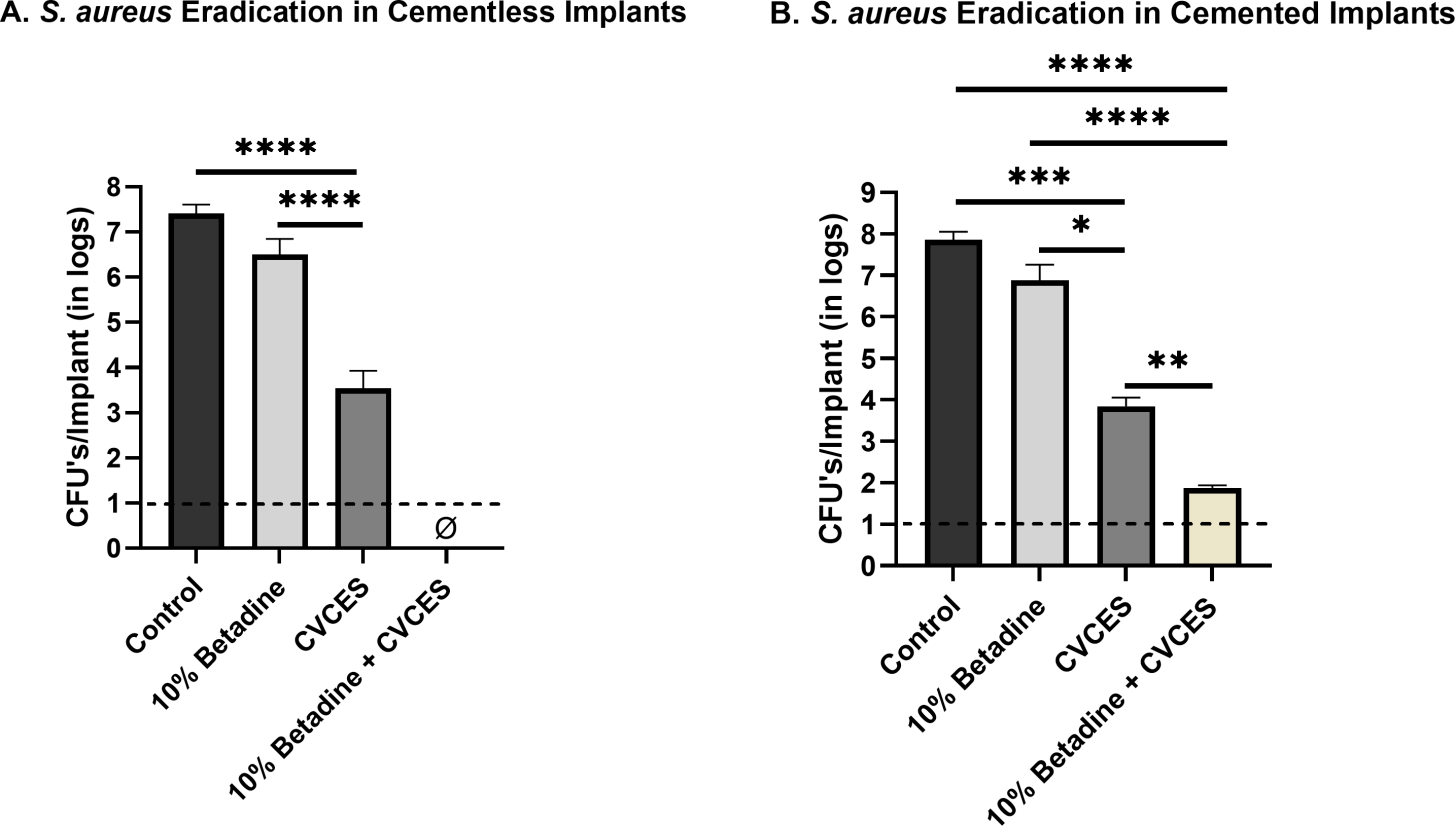
CFUs per implant of *S. aureus* biofilms enumerated from CoCr implants for control, exposure to 10% betadine, CVCES treatment, and combined treatment of 10% betadine followed by CVCES treatment. ɸ represents zero CFUs. A and B indicate recovered CFUs/implants in cementless and cemented patellofemoral implants, respectively. The limit of detection is denoted with a dashed line. Error bars indicate mean ± SEM. *P < 0.05, **P < 0.01, ***P < 0.001, and ****P < 0.0001.

The 48-hour biofilms of *S. aureus*, *P. aeruginosa*, and *E. coli* were treated with 10% betadine for 3 minutes followed by CVCES at −1.9V for 24 hours. In both cementless and cemented patellofemoral implants, CVCES alone showed an approximate 4 log reduction (*P* < 0.0001 and *P* = 0.0002), while 10% betadine alone had just 0.9 log reduction (*P* = 0.1599 and *P* = 0.1288) in *S. aureus* biofilms (Fig. 5). Complete elimination of cells was achieved with the treatment of 10% betadine + CVCES in cementless implants (*P* < 0.0001), whereas around 6 log reduction was found with 10% betadine + CVCES treatment in cemented implants (*P* < 0.0001). CVCES alone showed a 3 log reduction from 10% betadine in both cementless and cemented implants (*P* < 0.0001 and *P* = 0.0253).

**Fig 6:**
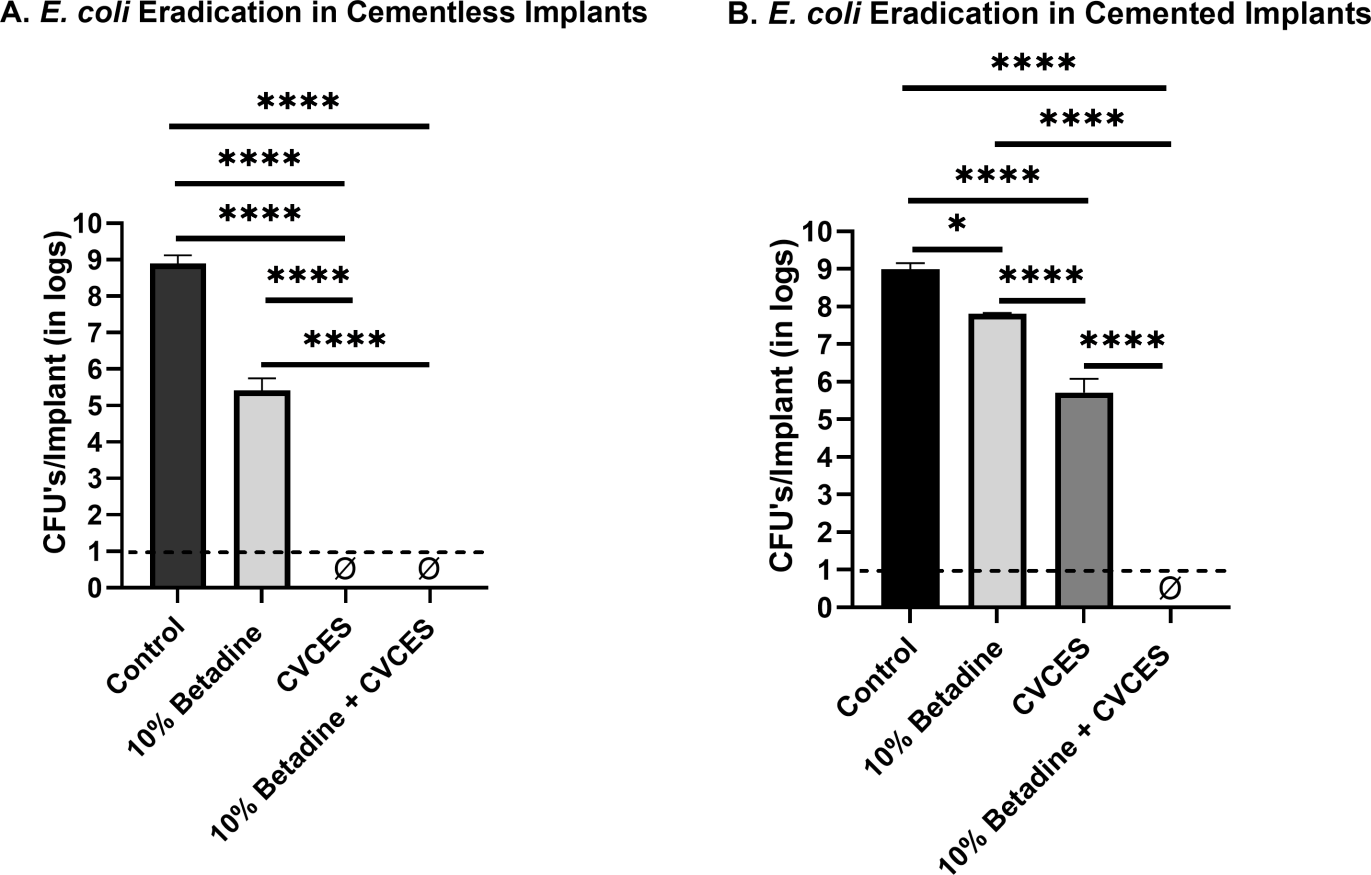
CFUs per implant of *E. coli* biofilms enumerated from CoCr implants for control, exposure to 10% betadine, CVCES treatment, and combined treatment of 10% betadine followed by CVCES treatment. ɸ represents zero CFU. A and B indicate recovered CFUs/implants in cementless and cemented patellofemoral implants, respectively. The limit of detection is denoted with a dashed line. Error bars indicate mean ± SEM. *P < 0.05; ****P < 0.0001.

In *E. coli*, complete killing was obtained in both cementless and cemented patellofemoral implants with 10% betadine + CVCES treatment (*P* < 0.0001). For the cementless implants, there was 3.48 log reduction (*P* < 0.0001) in the sample treated with 10% betadine (*P* < 0.0001), and no detectable colony was demonstrated with the use of CVCES alone (*P* < 0.0001) as presented in Fig. 6. Furthermore, 1.19 (*P* = 0.0484) and 3.27 log reductions (*P* < 0.0001) were found in cemented implants with the treatment of 10% betadine and CVCES, respectively. A 2 log difference (*P* < 0.0001) was observed between 10% betadine and CVCES treatment in cemented implants.

**Fig 7:**
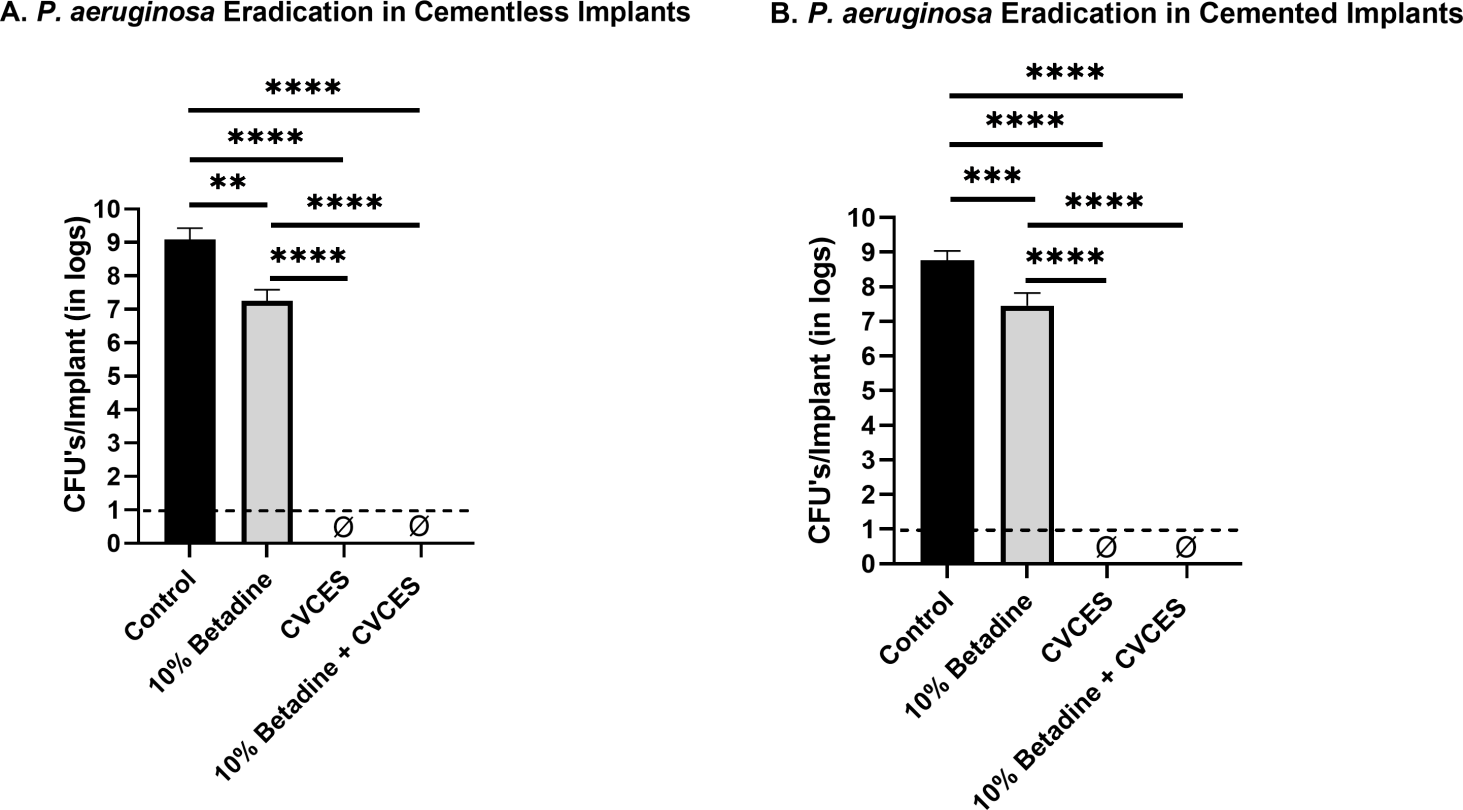
CFUs per implant of *P. aeruginosa* biofilms enumerated from CoCr implants for control, exposure to 10% betadine, CVCES treatment, and combined treatment of 10% betadine followed by CVCES treatment. ɸ represents zero CFU. A and B indicate recovered CFUs/implants in cementless and cemented patellofemoral implants. The limit of detection is denoted with a dashed line. Error bars indicate mean ± SEM. **P < 0.01, ***P < 0.001, and ****P < 0.0001.

In the case of Gram-negative organism, *P. aeruginosa*, the CFU results showed complete kill with CVCES and 10% betadine + CVCES treatment in both cementless and cemented patellofemoral implants (*P* < 0.0001). With 10% betadine, 1.83 (*P* = 0.013) and 1.31 (*P* = 0.0009) log reductions were achieved in cementless and cemented implants, respectively, as seen in Fig. 7.

### Eradication of biofilms using CVCES alone in commercially available femoral and tibial implants

**Fig 8:**
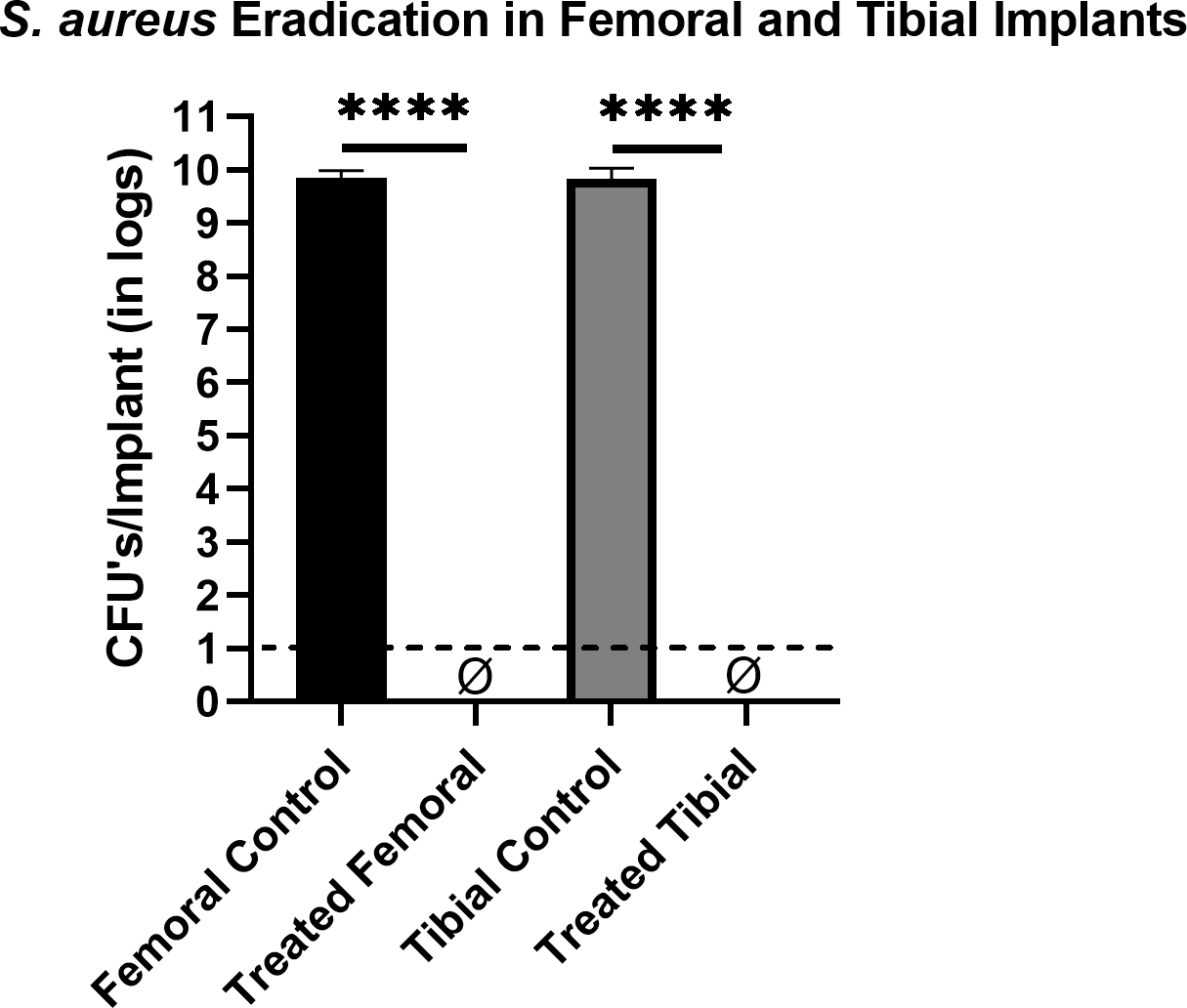
CFUs per implant of *S. aureus* biofilms enumerated from cementless femoral and tibial implants for controls and CVCES treatment. ɸ represents zero CFU. The limit of detection is denoted with a dashed line. Error bars indicate mean ± SEM. ****P < 0.0001.

The 48-hour biofilms of *S. aureus* were treated with CVCES at −1.9V for 24 hours on cementless commercially available femoral and tibial implants with the counter electrodes placed opposite to the reference electrode. CVCES treatment alone showed complete eradication of the bacteria on both femoral and tibial implants (*P* < 0.0001 and *P* = 0.0001) (Fig. 8).

### pH measurements and total charge transfer calculations after CVCES and 10% betadine + CVCES treatment

Increased pH and bacterial viability associated with CVCES have been shown to be related (26, 27); therefore, the pH was measured after treatment with CVCES and 10% betadine + CVCES. The results are shown in Table 1 for patellofemoral and femoral and tibial implants, respectively. The pH in all the treated samples was an alkaline pH of around 12, suggesting the increase in pH with the application of CVCES which might have led to the reduction in cell viability.

**TABLE 1:** Average pH measured following CVCES on patellofemoral implants

	Cementless		Cemented	
Bacterial species	CVCES	10% betadine + CVCES	CVCES	10% betadine + CVCES
*S. aureus*	12.54 ± 0.01	12.45 ± 0.08	12.50 ± 0.008	12.38 ± 0.01
*E. coli*	12.86 ± 0.06	12.94 ± 0.02	12.74 ± 0.06	12.75 ± 0.008
*P. aeruginosa*	12.80 ± 0.003	12.87 ± 0.05	12.82 ± 0.02	12.88 ± 0.001

Similarly, the current and the total charge transfers measured after each treatment are shown in Tables 2–4 for patellofemoral and femoral and tibial implants.

**TABLE 2:** Average current (mA) measured following CVCES on patellofemoral implants

	Cementless		Cemented	
Bacterial species	CVCES	10% betadine + CVCES	CVCES	10% betadine + CVCES
*S. aureus*	42.59 ± 0.96	36.49 ± 2.83	39.39 ± 6.30	33.13 ± 2.40
*E. coli*	49.76 ± 2.41	46.88 ± 5.50	46.13 ± 6.07	48.02 ± 0.20
*P. aeruginosa*	50.67 ± 7.07	57.28 ± 2.12	44.37 ± 1.90	46.59 ± 5.50

**TABLE 3:** Total charge transfer [Coulomb (C)] calculated following CVCES on patellofemoral implants

	Cementless		Cemented	
Bacterial species	CVCES	10% betadine + CVCES	CVCES	10% betadine + CVCES
*S. aureus*	3681.10 ± 83.700	3153.80 ± 245.05	3411.00 ± 546.06	2919.60 ± 153.85
*E. coli*	4300.10 ± 208.95	4051.60 ± 475.75	3988.80 ± 523.63	4151.20 ± 16.800
*P. aeruginosa*	4379.30 ± 610.67	4955.80 ± 178.05	3836.50 ± 162.48	4028.20 ± 474.70

**TABLE 4:** Average current (mA), total charge transfer [Coulomb (C)], and pH following CVCES on commercially available femoral (CoCr) and tibial implants (Ti)

Cementless			
Bacterial species	Current	Charge transfer	pH
*S. aureus*	269.25 ± 0.29	23267.20 ± 24.00	13.02 ± 0.006

### SEM imaging of the *S. aureus* and *P. aeruginosa* biofilms after treatment with CVCES and 10% betadine + CVCES in cementless patellofemoral implants

**Fig 9:**
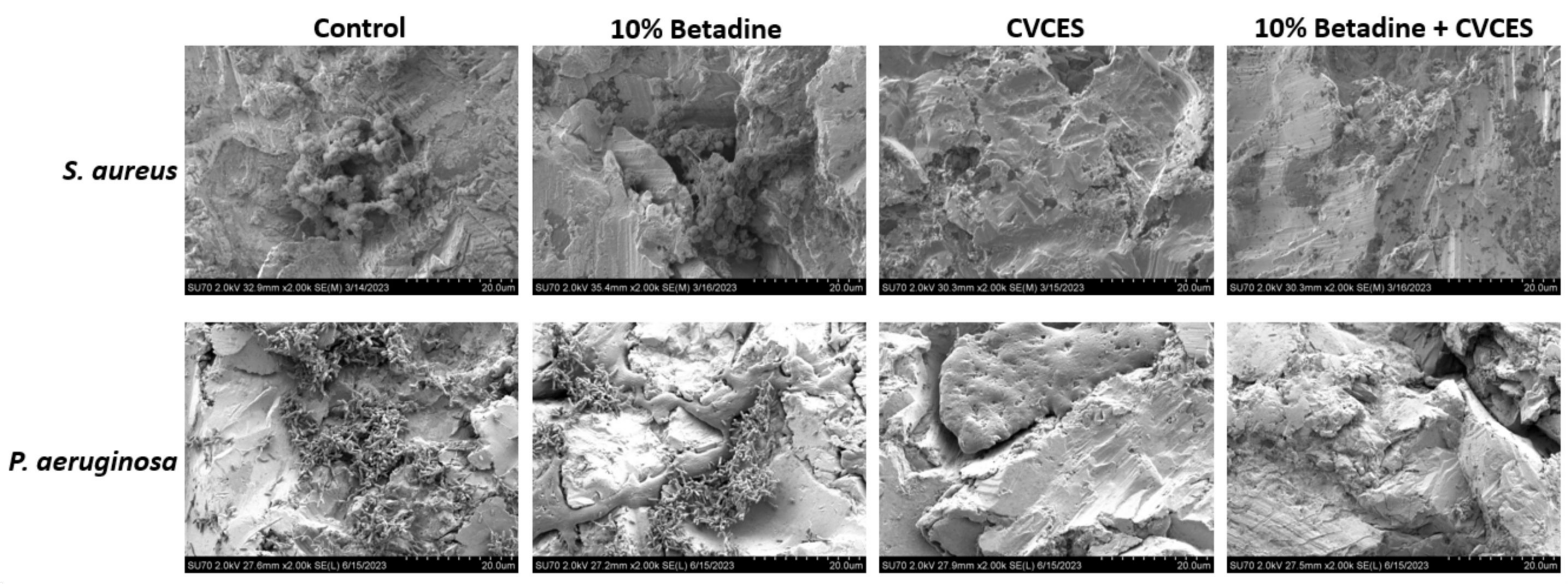
SEM images of bacteria on the CoCr surfaces of cementless patellofemoral implants at ×2,000 magnification of *S. aureus* and *P. aeruginosa* of the untreated and the treated implant surfaces.

Figure 9 shows the SEM images of *S. aureus* and *P. aeruginosa* on CoCr cementless patellofemoral implants. Groups of *S. aureus* and *P. aeruginosa* cells were observed on the surface that were not treated. The surface treated with 10% betadine also showed bacterial groups, like the untreated samples, with visible extracellular polymeric matrix binding the cells together. No bacteria were observed on the surface treated with CVCES and 10% betadine + CVCES compared to the controls of *P. aeruginosa*. However, the *S. aureus* biofilm treated with CVCES still had some viable bacteria on the surface, and zero cells were quantified when CVCES treatment was combined with betadine treatment.

## DISCUSSION

Currently, the treatment for PJI is often a two-stage revision arthroplasty involving removal of all components, placement of a temporary antibiotic spacer, and administration of intravenous antibiotics until eradication of the infection is confirmed with later revision to femoral components. This treatment strategy incurs both a financial burden to the healthcare system and increased patient morbidity. The removal of native bone and tissue with revision procedures along with long-term antibiotic therapy can have morbid consequences greatly affecting length and quality of life. Looking at the American healthcare system, a recent paper demonstrated that the per-case average cost of a revision total knee arthroplasty for infection in 2017 was $28,161 and the overall economic impact of PJI for both total hip and knee arthroplasties was $902 million (34). This is estimated to continue to increase. The use of alternative treatment methods is warranted especially with the emergence of multidrug-resistant pathogens. In the acute setting and with a stable prosthesis, a DAIR procedure is an accepted alternative treatment to a one- or two-stage revision arthroplasty. The ability to address the infection without removing implants and involving only a single operation makes this an attractive option, despite current published success rates ranging between 14% and 100% (14). The variability of success in this procedure is likely due to multiple factors: incomplete debridement of the biofilm from the arthroplasty component surfaces and surrounding tissues, ineffective irrigation, and the presence of cement that provides an additional surface with which the bacteria and associated biofilm may interdigitate and persist despite surgical debridement and antibiotic therapy. This study incorporates the use of a novel technology, CVCES, to be used alongside the DAIR procedure for the effective removal of biofilms from implant surfaces. While it has been used clinically, DAIR success rates vary widely. Due to the challenges in effectively treating implant infection with DAIR alone, CVCES would be applied to the implant after the DAIR procedure. This treatment creates a bactericidal electrochemical reaction, which produces hydrogen peroxide and hydroxide ions. The generation of these components raises the pH in a microenvironment around the implant, which kills the remaining bacteria that were left untreated by DAIR.

Our results with CVCES demonstrated complete eradication of the Gram-negative pathogens *P. aeruginosa* and *E. coli* from both cementless and cemented implants, whereas, in *S. aureus*, no detectable cells were found in cementless implants only. Most notably, the outcomes of this study reveal that a preliminary irrigation with betadine solution followed by CVCES treatment reduces biofilms from cementless implants to below detectable levels.

CVCES has been previously studied in both preclinical animal models and *in vitro* to prevent and treat orthopedic implant infections (26, 27, 32, 35–38). The initial work with this technology was conducted evaluating biofilm treatments on commercially pure Ti (cpTi) coupons. While Ti and its alloys are commonly used in orthopedic and dental applications due to their excellent biocompatibility and mechanical properties similar to bone, CoCr alloys are also often commonly used. It is important to note that CoCr was used as the primary material for the biofilm growth in this study due to its prevalence in total knee arthroplasty components. Few studies have been conducted regarding the biofilm formation on CoCr implants despite its widespread use in orthopedics (39). CoCr has a longer fatigue life (40) and has higher correction forces than Ti alloys (41), making it effective in orthopedic applications. In addition, CoCr is hard, tough, biocompatible, and one of the most commonly used metals in knee implants exclusively for femoral components (42). Additionally, the percentage of CoCr is high in most common orthopedic implants with 19% chromium in 316L stainless steel and 67% chromium in cobalt–chromium–molybdenum (43).

Previous research has been conducted on smooth surfaces that do not represent the implants *in vivo* as orthopedic implants have a wide range of surface features, textures, and roughness where biofilm can accumulate. Bacteria are more likely to attach to rougher surfaces due to increased surface area and depressions that provide more favorable sites for bacterial colonization via material surface irregularities (44, 45). To our knowledge, this is the first study demonstrating the efficacy of CVCES and betadine on biofilms formed on CoCr patellofemoral and commercially available femoral and tibial implants that represent different surface sizes, textures, and roughness found in clinical implants (46).

Our results from CFU assays show eradication to below detection limits of Gram-positive bacteria *S. aureus* in cementless implants after treatment with 10% betadine + CVCES. However, with CVCES only, incomplete kill was achieved in both cementless and cemented implants, suggesting that the bacterial structure may play a role in limiting CVCES treatment. In addition, there were still viable cells present after treating biofilms with 10% betadine + CVCES in cemented implants, suggesting the existence of more bioburden in the cemented implants vs the cementless implants.

The Gram-negative bacteria such as *P. aeruginosa* and *E. coli* demonstrated no detectable CFU after 24 hours of treatment in the absence of cement. In the presence of cement, complete eradication was achieved for both *P. aeruginosa* and *E. coli* when 10% betadine + CVCES was used. Notably, *P. aeruginosa* showed no detectable CFU with CVCES alone in the presence and absence of cement.

Oxygen and water reduction reactions are dominant at the potentials applied in this study. Hydroxide ion production as a result of these reactions increases the pH following treatment, which is thought to be responsible for the efficacy of the treatment used in this study (26, 27). This corroborates previous research showing the relationship between pH increase following electrical stimulation and bacterial viability (47–50). One study showed the correlation between alkaline pH and the application of cathodic stimulation of cpTi in the range of −1.0 V to −1.8 V vs Ag/AgCl while simultaneously demonstrating the prevention and eradication of MRSA and *Acinetobacter baumannii* biofilms (26, 27). Another study by Mohn et al. demonstrated the generation of an *in situ* alkaline environment around cathodic implants that reduce the *E. coli* viable cells by 2 orders of magnitude (47). Similar to previous studies, the pH in the present work also reached an alkaline pH value of 12 in all the tested conditions. The pH of 10% betadine is acidic (around 4), and it is still unclear what the exact mechanism of action pre-treatment with the 10% betadine plays in relationship to CVCES. This is an area of further research with not only this solution but also other commonly used irrigants in the treatment of PJI.

Hydrogen peroxide (H_2_O_2_) and hydrogen gas may also play a role in the antimicrobial effects of CVCES. H_2_O_2_ is the reactive oxygen species that is electrochemically generated at cathode surfaces and is shown to have antimicrobial effects (51–55). During the oxygen reduction reaction, the dissolved oxygen near the electrode is consumed, which generates hydroxyl ions and H_2_O_2_ (56). Oxygen reduction is then followed by water reduction upon applying sufficient cathodic stimulation that produces hydrogen gas and more hydroxyl ions (57) as explained by Clark (58). There was generation of H_2_O_2_ on a stainless steel surface after applying negative potential as shown by one study (59). Increasing the concentration of H_2_O_2_ toward the surface is what the current authors believe is treating bacterial biofilm growth (59).

In addition to the effects of H_2_O_2_ on bacterial growth, hydrogen gas was also suggested to independently have antimicrobial potential. One study from 2006 evaluated electrical current on *Staphylococcus epidermidis* biofilms (49). The increase in pH and hydrogen gas generation was anticipated to cause alkaline hydrolysis of the biofilm matrix with hydrogen gas bubbles affecting biofilm viability (49). In a more recent study, complete biofilm removal was achieved after the biofilm surface was treated with −1.5 V vs Ag/AgCl (60). Hydrogen gas was believed to be responsible for the biofilm removal by detaching the bacteria and biofilm matrix from the surface as suggested by the authors (60).

In the presence and absence of cement, there was a high number of surviving cells after exposure to 10% betadine alone. This suggests that 10% betadine treatment alone may not decrease the bioburden enough on the implants to effectively treat PJI. The addition of CVCES, which creates an increase in pH and charge transfer across the implant surface, is an effective adjuvant. The current and charge transfer measured after CVCES ranged from 40 to 50 mA and 2,000–5,000 C, respectively. There was no significant difference in the current as well as charge transfer measured between groups. However, across treatment groups, when the average charge transfer was greater than 4,000 (average current 47 mA), there were no surviving CFUs detected.

The presence of cement was observed to influence the effectiveness of both the 10% betadine wash and CVCES in decreasing the CFU colonies to below detectable limits. In the case of *E. coli*, CVCES treatment did not decrease the CFU count as effectively in the presence of cement vs without cement even though the current and pH generation were similar. Similarly, in *S. aureus*, despite current being almost the same, the combination of 10% betadine and CVCES with the cemented implant had 10^2^ CFUs/implant, while no colonies were detected in the same treatment group with cementless implants. This suggests that there are likely retained bacteria within the pores of the cement not treated by the CVCES or 10% betadine wash. While CVCES effectively treats the metal surface of the implant, its effects do not appear to persist in the nonconductive cement. It is well known that bacteria prefer to attach to rougher surfaces and are able to penetrate the pores of the cement, which is a potential contributing factor to the differences in growth and CVCES response between cemented and cementless implants.

The SEM images in the current study depict the efficacy of the surface treatment of the biofilms on the cementless implants. The biofilm was intact and clustered together with a visible matrix on the controls and betadine-treated sample. The treatment with 10% betadine + CVCES resulted in more biofilm destruction as compared to the controls and 10% betadine alone treated sample in both *S. aureus* and *P. aeruginosa*. No cells were observed in the CVCES-treated samples with *P. aeruginosa* as shown by SEM images, while some surviving cells were still present in *S. aureus* by CFU’s assay. Because of the presence of low bioburden, SEM could not spot the cells in the CVCES-treated *S. aureus* biofilm. Therefore, full eradication of biofilm was achieved only with the combinatorial treatment of 10% betadine and CVCES in the case of *S. aureus* as there were no detectable CFUs based on quantitative assay. The absence of appreciable surviving bacteria after the CVCES treatment suggests the potential of this method for future clinical applications. On the other hand, *E. coli* treated with CVCES had surviving cells on the cemented implants as per the CFU results. However, due to the limitation of the microscope, imaging the cemented implants was not possible. Furthermore, *P. aeruginosa* was selected for representative Gram-negative biofilm infection due to it being more pathogenic than *E. coli*. Hence, the combinatorial treatment of betadine and CVCES in *E. coli* could be a promising antimicrobial approach when used in conjunction with cemented implants.

We acknowledge some limitations of this study. CFU enumeration used in this study accounts only for viable cells. The addition of microscopic examination of the surface with confocal microscopy would assess all cells, living and dead. Also, understanding the role of reactive oxygen and nitrogen species, hydrogen peroxide, pH, current, and charge transfer and their role in bacterial kill is another limitation and future consideration of this study. It is also important to understand how the different-sized implants and implant materials will affect the total charge transfer/current over the entirety of a CVCES treatment clinically. Additionally, considering the histology of the joint space will be an important measure to ensure that the electrical stimulation is not causing harm to the surrounding tissues. Finally, the results of this study are also only applicable *in vitro*. Currently, *in vivo* studies are being undertaken to examine the safety of this type of treatment as well as the efficacy of it being carried out with an already implanted device at various lengths of stimulation.

This work herein presents the potential application for the use of CVCES in the eradication of common pathogens found in PJI. This novel technology could be used along with DAIR treatment as an adjuvant to the current standard of care.

### Conclusion

The obtained results provide evidence of the eradication of biofilm with the use of CVCES alone and 10% betadine + CVCES treatment on orthopedic implants in common bacteria during PJI. The work presented reveals a promising development in treating PJIs, offering an alternative to a one- or two-stage exchange, which is often considered the current standard of care. While we acknowledge that future studies will be necessary to fully understand the mechanism of biofilm removal from the surfaces, our study provides evidence of biofilm removal with the use of novel technology and may provide effective treatment strategies to prevent and eradicate PJIs in the future.
